# Comparison of the Clinical Course, Management and Outcomes of Acute Pancreatitis in Aged and Young Patients

**DOI:** 10.3390/biomedicines14010139

**Published:** 2026-01-09

**Authors:** Agnieszka Krajewska, Katarzyna Tłustochowicz, Adrianna Kowalik, Ewa Małecka-Wojciesko

**Affiliations:** Department of Digestive Tract Diseases, Medical University of Lodz, ul. Kopcinskiego 22, 90-153 Lodz, Poland; katarzyna.tlustochowicz@stud.umed.lodz.pl (K.T.); adrianna.kulis@stud.umed.lodz.pl (A.K.)

**Keywords:** acute pancreatitis, elderly patients, age-related differences, clinical outcomes, mortality, geriatric patients, disease severity

## Abstract

Acute pancreatitis (AP) is an inflammatory condition with varying severity, ranging from mild self-limiting episodes to life-threatening complications. The incidence, clinical presentation, and outcomes of AP differ significantly across age groups, with elderly patients demonstrating distinct challenges. Biliary pancreatitis is more prevalent in older adults, whereas alcohol-induced AP dominates in younger populations. Elderly patients frequently present with atypical or less pronounced abdominal symptoms, which may delay diagnosis. Comorbidities such as kidney failure, cardiovascular disease, diabetes mellitus and arterial hypertension are significantly more common in the elderly and are associated with increased risk of organ dysfunction, systemic complications such as organ failure, multiple organ dysfunction syndrome (MODS), and prolonged hospitalization. The higher incidence of intensive care unit admissions and mortality is noted in the elderly, particularly in those over 80 years, in particular. Evidence on age-related differences in local pancreatic complications is inconsistent, with a possible trend toward lower rates in older adults. Early identification and individualized treatment planning are essential. Abundant fluid administration should be limited in older patients due to frequent cardiac insufficiency but should be carefully monitored due to the present or threatening renal insufficiency. Pain control with opioids may cause severe CNS complications for elderly patients. In contrast, ERCP, when indicated, is usually well tolerated in older patients. Personalized management in elderly patients is strongly recommended.

## 1. Introduction

Acute pancreatitis (AP) is an acute inflammatory condition of the pancreas that may involve peripancreatic tissues and distant organ systems. The clinical course of AP ranges from mild self-limiting illness to a severe form with systemic inflammatory response, multiorgan failure, and potentially fatal outcomes [1]. Numerous studies have demonstrated that the incidence of AP rises significantly with age. A large population-based cohort study conducted in Republic of Korea reported age-specific incidence rates of acute pancreatitis as follows: 16.30 per 100,000 person-years for individuals aged 20–39 years, 27.85 for those aged 40–64 years, and 57.19 for those aged 65 years and older [2]. Additionally, population-based analysis from the Global Burden of Disease (GBD) provided valuable insights into the global, regional, and national burden of acute pancreatitis (AP) across 204 countries and territories over a 30-year period (1990–2019). The analysis demonstrated that the incidence rate of AP (per 100,000) was highest among individuals aged >70 years across all regions and age groups. Elderly individuals (≥65 years) also carried a disproportionately high burden of AP-related mortality, accounting for 45.7% of all global deaths from AP in 2019. Although age-standardized death rates (ASDR) showed a modest decline, the absolute number of deaths and disability-adjusted life years (DALYs) continued to increase among older adults [3]. Elderly patients often present different physiological responses and a higher burden of comorbidities such as kidney failure, cardiovascular disease, diabetes mellitus and arterial hypertension compared to younger individuals [4,5].

In this review, “elderly,” or “older patients,” primarily refers to those aged ≥65 years, while “very elderly” or “oldest old” refers to patients aged ≥80 years, when such an age group was specifically reported in the studies. In some studies, however, elderly patients were defined as those aged >60 years. All these differences are described in the corresponding sections of the manuscript.

## 2. Etiology of AP Across Age Groups

In the general population, the most frequent causes of AP, listed in descending order of prevalence, include cholelithiasis, excessive alcohol consumption, hypertriglyceridemia (typically >1000 mg/dL), drugs (e.g., thiopurines, asparaginase, didanosine, valproic acid, mesalamine), post-endoscopic retrograde cholangiopancreatography (post-ERCP), abdominal trauma, postoperative complications, autoimmune pancreatitis (types 1 and 2), and infections such as cytomegalovirus, mumps virus, Epstein–Barr virus, or *Toxoplasma gondii* [6].

Age-related differences in AP etiology have been well evaluated. In a prospective study, patients with AP were divided into age groups: 18–64 years (Group I), 65–79 years (Group II), and ≥80 years (Group III). Cholelithiasis was the predominant etiological factor among older patients, accounting for 54.1% and 58.1% of cases in Groups II and III, respectively, compared to 22.5% in Group I (*p* < 0.000). Conversely, alcohol-related AP was significantly more prevalent among younger individuals (36.2% in Group I) and rare in the elderly (4.1% in Group II and 0% in Group III) (*p* < 0.000). The proportion of idiopathic cases did not differ significantly between age groups [7].

Similar findings were reported in a retrospective analysis of 1150 patients: biliary pancreatitis was the leading etiology across the entire population (<65 years: 47.6%; 65–80 years: 72.2%; ≥80 years: 89.6%), respectively. Alcohol-related AP occurred in 10.2% patients under 65 years, 9.5% patients from 65 to 80 years and 0.9% patients over 80 years old. Hypertriglyceridemia was significantly more common in patients under 65 years (14.3%) than in those aged 65–80 years (1.2%) or ≥80 years (0%). Idiopathic AP was the second most frequent cause in patients over 80 years (6.6%) and the third most frequent in the younger groups: <65 years (11.9%) and 65–80 (5.3%) [4].

The results were further supported by a large retrospective study encompassing 5652 patients, which showed that hypertriglyceridemia accounted for 39.5% of AP cases in patients <60 years, compared with only 5.6% of cases among patients ≥60 years. In patients <60 years, idiopathic AP and biliary etiologies comprised 21.9% and 19.3% of cases, respectively, whereas, in patients aged ≥60 years, biliary etiology was dominant (55.2%), followed by idiopathic causes (22.8%) [8].

According to the cited literature, gallstone-related pancreatitis clearly predominates in older patients (≥65 years), whereas alcohol and hypertriglyceridemia are more frequent triggers in younger adults. This distribution likely reflects age-related differences in lifestyle and metabolic risk profiles, as younger individuals tend to have higher rates of alcohol consumption, including binge drinking, which may also contribute to elevated triglyceride levels [9]. Together, this findings highlight a clinically important shift in etiology with aging.

## 3. Age-Related Clinical Presentation and Course of AP

### 3.1. Symptoms and Signs

The symptoms and clinical course of AP in elderly patients differ from those observed in younger individuals—not only in terms of symptom type and intensity but also due to the presence of comorbidities and higher mortality rates. In older adults, the symptoms of AP are often less pronounced than in younger patients.

Çalim et al. conducted a retrospective study involving 500 patients aged over 65 years diagnosed with AP. Patients were divided into three groups: 65–74 years, 75–84 years and ≥85 years. Across all the groups, the most common symptoms were abdominal pain, nausea, and vomiting [10]. However, abdominal pain was significantly less frequent in the oldest group (≥85 years), occurring in 89.8% of patients, compared with 97.7% in the 65–74 age group and 96.1% in the 75–84 age group (*p* = 0.006). This difference may be explained by the age-related factors such as neuropathy, increased pain tolerance, or perineurial fibrosis. On the other hand, fever was significantly more prevalent in the ≥85 years group (*p* = 0.012).

In another retrospective study by Yu et al. involving 473 patients with AP, abdominal pain was more frequent in the 60–80-year age group (61.3%) compared to those over 80 years (46.3%) [11]. Additionally, symptoms such as jaundice (17.5% vs. 8.9%, *p* = 0.021) and dyspnoea (26.3% vs. 11.5%, *p* = 0.001) occurred more often in patients over 80 years old. The higher prevalence of dyspnoea in this group may be explained by reduced cardiopulmonary reserve, frailty, and a higher burden of comorbidities, while jaundice likely reflects the predominance of biliary etiology, delayed presentation and decreased hepatic reserve in the oldest group. The incidence of vomiting was similar between the older and oldest groups (6.6% vs. 10.0%) [11].

### 3.2. Laboratory Profiles

Several studies have reported age-related differences in laboratory findings among patients with AP groups. CRP levels in elderly patients may be lower due to age-related reductions in immune response, which can mask the true extent of inflammation. Haemoglobin and albumin concentrations are also lower in older patients than younger patients (*p* < 0.001). These findings are clinically relevant, as hypoalbuminemia and anaemia are common in the geriatric population and often reflect underlying comorbidities and nutritional deficits [12]. Conversely, mean serum amylase levels were significantly higher in the elderly compared with younger patients (*p* < 0.05) [11]. However, this finding has limited clinical value. Serum amylase alone has insufficient predictive power for determining the severity or etiology of AP, and elevated levels do not always confirm the diagnosis. Current evidence shows that up to 10% of all patients with elevated amylase may not have AP. Therefore, amylase levels in elderly patients should always be interpreted in conjunction with clinical presentation and imaging to avoid overdiagnosis and unnecessary interventions [7].

## 4. Comorbidity Burden and Systemic Complications

Elderly patients with acute pancreatitis exhibit a higher burden of comorbidities and are more likely to develop a systemic inflammatory response, organ failure, and multiple organ dysfunction syndrome (MODS) compared with younger individuals. The oldest patients with AP (≥80 years) more frequently than younger patients present with arterial hypertension (55–73%), cardiovascular disease (38–42%), diabetes mellitus (17–46%), heart failure (18%), atrial fibrillation (11%), osteoarthritis (3%), chronic obstructive pulmonary disease (COPD) (8–33%), and chronic kidney disease (4–15%) [4,7]. A retrospective study by Kalkan et al. examined 1150 AP patients and found that comorbidities were significantly more prevalent in elderly patients aged 65–80 years and those over 80 years compared to those under 65 years [4]. Specifically, cardiovascular, pulmonary, and renal comorbidities were more common in the elderly. In patients over 80 years, the prevalence of diabetes was 46.2% and hypertension was 73.6%, compared to 11.8% and 13.6%, respectively, in those under 65 years [4]. Pleural effusion was also more frequent in patients over 80 years (28.3%) compared with those aged 65–80 (10.6%) and under 65 years (1.4%). MODS was observed in 9.42% of patients over 80 years, compared to 0.6% in those under 65 years.

A study by Zhang et al. included 756 elderly (>60 years) and 4896 younger (<60 years) AP patients [8], a higher incidence of persistent respiratory (23.7% vs. 17.9%, *p* < 0.001) and cardiovascular failure (4.5% vs. 2.9%, *p* = 0.015) was observed in the elderly group. Moreover, older patients had increased rates of persistent organ failure (OR: 1.623, 95% CI: 1.326–1.987, *p* < 0.001) and multiple organ failure (MOF) (OR: 1.757, 95% CI: 1.186–2.604, *p* = 0.005). According to another large retrospective cohort of patient with first-attack of AP (*n* = 2965), patients >60 years had more cardiovascular/cerebrovascular comorbidities and higher rates of MODS compared with younger adults [13].

## 5. Local Complications

Reports on age-related differences in local complications are inconsistent. According to the findings of Kalkan et al., a higher rate of local complications was reported among older patients, with acute peripancreatic fluid collection observed in 41.7% of those aged 65–80 and 50.9% of those over 80 years, compared to 27.2% in those under 65 years. Acute necrotic collection and walled-off necrosis were more frequent in the elderly, with 12.4% in the 65–80 age group and 36.8% in those over 80 years, compared to 1.6% in those under 65 years. In contrast, pancreatic pseudocyst rates did not statistically differ between the groups [4]. Interestingly, a retrospective study of 169 patients diagnosed with severe acute pancreatitis (SAP), who were stratified into two cohorts based on age: ≥60 years and <60 years showed that the extent of local pancreatic injury on imaging, reflected by the CT severity score was significantly higher in the younger group (4.8 ± 1.8 vs. 4.0 ± 1.9, *p* = 0.0020). These findings suggest that, on average, local pathological changes were less severe ≥60 years patients than in younger individuals. However, to interpret these results with caution and to account for potential heterogeneity within the older population, the authors further analyzed outcomes among older survivors and non-survivors. Notably, CT severity scores were significantly higher in deceased ≥60 years patients, indicating that CT-based assessment remains a sensitive predictor of mortality in severe acute pancreatitis [14]. Pancreatic abscess occurred more often in the younger group (17.3%) compared with (13.9%) in the older group [14]. Similarly, the incidence of other local complications, including pseudocysts and walled-off necrosis, did not significantly differ between age groups. These findings suggest that despite the higher physiological severity reflected by APACHE II and Ranson scores, observed in older patients, local pancreatic damage on imaging (CT severity score) was less pronounced in older compared to younger patients.

Similarly, Zhang et al. compared elderly patients (>60 years) with younger ones (<60 years), and found that elderly patients exhibited a lower incidence of local complications, including acute peripancreatic fluid collection (34.4% vs. 41.0%, *p* < 0.001) and acute necrotic collection (6.6% vs. 10.0%, *p* = 0.003) [8]. This finding is noteworthy because, despite a significantly higher burden of systemic complications, a parallel increase in local complications was not observed. The reduced frequency of local complications in elderly patients could potentially be explained by age-related differences in the inflammatory response and pancreatic tissue reaction to injury, resulting in reduced peripancreatic fluid collections or necrosis visible on imaging [8]. Another possible explanation is a different clinical course of AP in elderly patients, in whom systemic inflammatory response predominates over local tissue damage [8]. Another possible hypothesis is that, as a consequence of age-related changes, pancreatic exocrine function becomes impaired, which may in turn lead to a lower incidence of local complications. However, this hypothesis requires further investigation [15]. Together, these data support the hypothesis that systemic inflammatory response, rather than local pancreatic damage, plays a predominant role in driving poor outcomes and higher mortality in the elderly.

## 6. Mortality and Predictors of Poor Outcomes

Comorbidities seem to be associated with a more severe course of AP and higher overall mortality in elderly patients [16]. The overall mortality rate associated with acute pancreatitis (AP) is estimated to range between 2% and 5% worldwide [16]. However, accumulating evidence indicates a strong age-related gradient in mortality. A 2019 meta-analysis of 33 studies including 194,702 patients with AP demonstrated a linear increase in mortality with advancing age, particularly after the age of 59. The mortality rate was as low as 0.9% among individuals under 20 years of age, but rose by 0.086% annually between ages 20 and 59, and by 0.765% annually between 59 and 70, culminating in a nine-fold increase by age 70 and a 19-fold increase in patients over 70 compared to those under 20. This association was statistically confirmed with meta-regression analysis. Disease severity also emerged as an independent predictor of mortality [5].

An increased mortality among older patients was observed with an overall mortality rate of 9.9%. The mortality rate rose to 15.7% was observed in patients >65 years, and a dramatic increase in mortality to 50% was observed in patients aged ≥ 80 [4]. In the latter group, mortality was significantly associated with prolonged hospital stay of the hospital duration of 15.51 ± 8.25 compared to 5.58 ± 3.44 in patients under 65 years. Multivariate analysis identified several independent predictors of death in patients ≥80 years, including moderate to severe pancreatitis, systemic and local complications, major polypharmacy, elevated serum creatinine (>2.4 mg/dL), and high C-reactive protein levels (>40 mg/dL) [4]. In a cohort by Zheng, mortality in younger patients declined steadily after the initial peak, dropping to 7.69% by week five, compared with 13.33% in the elderly group. The higher mortality observed in elderly patients seems to be linked to single organ failure (SOF), MOF, and infections [8].

## 7. Age-Related Severity Assessment and Healthcare Utilization

### 7.1. Risk Stratification Scores

Age is considered a critical factor in predicting the severity of AP, reflected in commonly used scoring systems, including APACHE II, BISAP score, Ranson criteria, and the Revised Atlanta Classification. The cut-off points vary between age 45 and 60 years across different scoring systems, reflecting different epidemiological data and demographics. Nevertheless, they all reflect the poorer prognosis connected with advanced age [16]. Several studies consistently show the connection between older age and higher severity scores and worse outcomes.

Large retrospective cohorts illustrate the gradient in severity related to age. In a retrospective study of 1150 patients with AP, participants were categorized into three age groups. The severity of AP in each group was assessed based on the Revised Atlanta Criteria [17]. In patients under 65 years of age (*n* = 706), the majority experienced mild course of AP 72.1%. Moderately severe AP was observed in 25.8% of patients, and only 2.1% had severe AP. In the intermediate age group (65–80 years), 39.9% had mild AP, 45.3% had moderately severe AP and 14.8% had severe AP. Conversely, in patients over 80 years old (*n* = 106), mild AP was observed only in 13.2% patients, while 47.2% had moderately severe AP, and 39.6% experienced severe course of AP [4]. Therefore, age over 80 represents a particularly strong factor for poor AP prognosis.

Similarly, a separate retrospective study of 602 patients with AP divided into two groups, found that severe AP (according to the revised Atlanta) occurred in 8.7% of the older group (over 65 years), compared to only 1% of severe AP in younger group (under 65 years), underscoring the disproportionate burden of severe disease among older adults. The APACHE II score also seems to mirror this trend. In a retrospective study by Yu et al., 473 patients with AP were divided into two groups: aged (60–80 years) and (≥80 years) [11]. The mean APACHE II score in the oldest group (13.08 ± 3.50) was significantly higher than the score of the aged (60–80) (11.9 ± 3.7) (*p* = 0.01).

Similar findings were shown in a nationwide Polish study conducted in 2018 by Koziel et al. in which 963 AP patients were divided into three groups (18–64 years, 65–79 years and over 80 years) [7]. The assessment of severity using the APACHE II scale showed that older patients had significantly higher mean scores with values of 7.49 in 65–79 group and 8.99 in the oldest group, respectively. On the other hand, patients aged under 65 years had lower values of 3.88 as the mean score [7]. In a retrospective cohort study, 169 patients diagnosed with severe acute pancreatitis (SAP) were stratified into two cohorts based on age: ≥60 years and <60 years. The results demonstrated significantly higher APACHE II and Ranson scores in the elderly group compared to younger patients (3.4 ± 1.7 vs. 2.8 ± 1.6, *p* = 0.0069). Across retrospective and prospective cohorts, patients aged ≥65 years with AP consistently demonstrated higher severity scores (Ranson at admission and 48 h, BISAP, APACHE II) and worse outcomes than younger adults [11,18,19].

### 7.2. Length of Hospital Stay

Older age is associated with longer hospital stays and a higher mortality rate (9.6% vs. 0.5%) [12], compared with the younger patients. Even among those with mild AP, patients above 65 years of age had significantly longer hospitalizations than their younger counterparts [7], likely due to comorbidities such as heart failure, chronic obstructive pulmonary disease and atherosclerotic heart disease. Additionally, patients ≥80 years had significantly longer hospitalizations compared to those aged 60–80 years (11.51 ± 10.19 vs. 8.70 ± 6.56 days; *p* = 0.003) [11]. Together, these findings indicate that advanced age, particularly ≥80 years, is associated with prolonged hospitalization. Although some studies have suggested that shorter hospital stays observed in elderly patients with severe AP may reflect early in-hospital mortality rather than early recovery or discharge, Yu et al. did not report such an association directly [11]. Their data primarily demonstrate that hospitalization duration tends to be longer in the oldest patients, likely reflecting the complexity of care and comorbidity burden.

### 7.3. ICU Admissions and Critical Care Outcomes

Elderly patients diagnosed with AP seem to exhibit higher rate of hospitalization in ICU compared to the younger populations. This dramatic increase in ICU admission was observed in study by Kalkan, where only 2.5% (18 patients) of aged under 65 required ICU admission, compared with 18.6% (63 patients) aged 65–80 and 49.1% (52 patients) aged ≥ 80 years) [4].

This data are consistent with findings from the study by Yu, in which two groups: 393 patients in older group (60–79 years) and 80 in oldest (over 80 years) of elderly patients with AP were compared. In both groups, ICU admissions rates were high, with 32.8% among older group and 37.5% in the oldest group. BMI (*p* = 0.001), SAP, a more severe AP presentation (*p* = 0.001), hypertriglyceridemia aetiology (*p* = 0.026), absence of typical AP symptomatology of abdominal pain (*p* = 0.001), necrosectomy (*p* = 0.018), acute respiratory failure (*p* = 0.001) and local complications (*p* = 0.001) were significantly associated with a higher ICU admission rate in the univariate analysis. However, in the multivariate analysis, ICU admission was associated with a more severe AP presentation (*p* = 0.001), the absence of abdominal pain (*p* = 0.033), acute respiratory failure (*p* = 0.001), and multiorgan failure (*p* = 0.022) [11].

Additionally, a study of 218 patients with AP with median age of the 76 years and age range from 65 to 113 years was conducted. Of these patients, 16 (7.3%) were directly admitted to the ICU, while 197 (90.4%) were admitted to the general ward. Statistically significant differences were observed in neutrophil count at 48 h (*p* = 0.004), lymphocyte count at 48 h (*p* = 0.009), and NLR at 48 h (*p* < 0.001) between patients admitted to the general ward and those admitted to the intensive care unit. This data also indicates a higher tendency for ICU admission in elderly; however, the observed rate is lower than reported in previous studies, which may result from excluding patients subsequently transferred to the ICU from other wards and not directly admitted to the Emergency Department [20].

The key findings from Section 2, Section 4, Section 6 and Section 7 are summarized in Table 1.

## 8. Management Considerations in Elderly Patients

### 8.1. Fluid Resuscitation

Fluid therapy in acute pancreatitis should be individualized according to disease severity, patient age, comorbidities, and overall clinical status. According to the latest ACG guideline, lactated Ringer’s solution is recommended over normal saline for intravenous resuscitation in AP [22]. The latest clinical trials comparing moderately aggressive intravenous hydration (approximately 1.5 mL/kg/h) with more aggressive regimens (e.g., 3 mL/kg/h) suggest that moderate fluid therapy appears to be associated with more favorable outcomes than higher-volume infusion [23].

According to a narrative review of the literature, a stratified approach has been proposed: lower infusion rates and total fluid volumes for mild acute pancreatitis (e.g., approximately 1.5 mL/kg/h with a bolus of 10 mL/kg over 1–2 h in patients with hypovolemia), and higher rates or more aggressive resuscitation for moderate-to-severe cases (e.g., 1.5–3 mL/kg/h with a bolus of 10–20 mL/kg over 1–2 h, or higher in hypotension), guided by clinical and laboratory parameters [24].

In older patients or those with significant comorbidities, particularly cardiac or renal dysfunction, fluid resuscitation should be performed with caution due to the increased risk of volume overload. Close and continuous monitoring is essential, including heart rate, mean arterial pressure, urine output, hematocrit, blood urea nitrogen, creatinine, oxygen saturation, and, when available, bedside ultrasound assessment of the inferior vena cava or cardiac filling. Early signs of fluid overload—such as pulmonary crackles, weight gain, decreased oxygenation, or peripheral edema—should prompt immediate reduction or cessation of fluid infusion. If volume overload develops, diuretics may be administered in patients with preserved renal function, whereas those with refractory congestion or oligoanuria should be considered for early renal replacement therapy [25].

### 8.2. Pain Management

Pain management in older patients generally follows the same principles as in the younger population and is guided by the World Health Organization (WHO) analgesic ladder [26]. However, there are specific challenges due to concomitant medication use and impaired kidney and liver function. Acetaminophen is considered a first-line analgesic option for mild to moderate pain in older patients, particularly in those with contraindications to NSAIDs, such as chronic kidney disease, peptic ulcer disease, or increased bleeding risk. Owing to its favorable gastrointestinal and renal safety profile, paracetamol may be used as monotherapy in mild pain or as part of a multimodal analgesic strategy in combination with opioids for more severe pain. In older adults, the recommended maximum daily dose should generally not exceed 4 g/day. However, in individuals with low body weight, malnutrition, liver disease, or chronic alcohol use, a reduced maximum dose of 2000–3000 mg per day is advisable to minimize the risk of hepatotoxicity [26].

The American Geriatrics Society recommends that NSAIDs should be used with great caution in older adults, only when necessary, at the lowest effective dose and for the shortest possible duration—not exceeding 10 days—due to the increased risk of gastrointestinal bleeding, kidney injury, and cardiovascular adverse events in this population [26].

NSAIDs with a shorter half-life, such as dexketoprofen, ketoprofen, diclofenac, or ibuprofen, are preferred to minimize cumulative toxicity. The risk of NSAID-induced gastrointestinal bleeding is three- to six-fold higher in adults aged ≥65 years than in younger individuals [27], primarily owing to impaired mucosal healing, mucosal ischemia, multimorbidity, concomitant anticoagulant therapy, and age-related pharmacokinetic changes [27]. In a study by Tian-Yu et al. involving 4728 patients over 60 years of age who were taking NSAIDs, gastrointestinal bleeding occurred in 928 individuals. The primary risk factors included a family history of gastrointestinal bleeding, history of peptic ulcers, cardiovascular and cerebrovascular diseases, diabetes mellitus, use of antiplatelet agents, *Helicobacter pylori* infection and NSAID use for a duration of 0.5 to 3 months [28]. In patients at high risk of gastrointestinal bleeding—including those over 75 years old, with a history of peptic ulcer disease or prior gastrointestinal haemorrhage, and those receiving anticoagulants, antiplatelet agents, or corticosteroids—NSAIDs should generally be avoided [29]. If NSAID therapy is required, selective COX-2 inhibitors in combination with a proton pump inhibitor may be considered to reduce the risk of gastrointestinal bleeding [29].

NSAIDs differ in their cardiovascular risk profile. Diclofenac, high-dose ibuprofen and selective COX-2 inhibitors are associated with a higher incidence of cardiovascular events and exacerbation of heart failure [30]. In patients with significant cardiovascular disease—particularly those with heart failure or a recent myocardial infarction—these agents should be avoided. Instead, analgesic options with a more favourable cardiovascular safety profile, such as naproxen should be administered [31]. NSAIDs are nephrotoxic, particularly ibuprofen, naproxen and diclofenac, due to their inhibition of renal prostaglandin synthesis, which can reduce renal perfusion and precipitate acute kidney injury, especially in older adults and in the setting of acute pancreatitis. In elderly patients with chronic kidney disease or significantly reduced glomerular filtration rate, these drugs should generally be avoided or used only short-term with close monitoring of renal function [32]. Weak opioids such as tramadol, may be considered for moderate pain unresponsive to other treatments. However, tramadol can lower the seizure threshold and should be avoided in patients with a history of seizures or epilepsy. Additionally, it should not be combined with serotonergic antidepressants—including TCAs, SNRIs, or SSRIs—due to the risk of serotonin syndrome [33]. Older adults are at heightened risk for more severe opioid-related adverse effects, including sedation, constipation, nausea, urinary retention, and falls secondary to orthostatic hypotension, depression, anxiety, psychosis with respiratory depression representing the most serious and potentially life-threatening complication [34]. Consequently, it is generally recommended that opioid therapy be initiated at low doses and carefully increased if needed, with initial doses reduced by 25–50% compared to those used in younger individuals, followed by cautious titration based on clinical response and side-effect profile [35]. Hydromorphone, oxycodone, fentanyl (short-acting) are recommended for acute severe pain, as they have shorter half-lives and pose less risk of prolonged sedation or accumulation compared to long-acting forms [35].

### 8.3. Nutritional Care in the Elderly

Older adults with acute pancreatitis are at moderate–to–high risk of malnutrition because AP is highly catabolic and older patients more often have pre-existing frailty, comorbidity and sarcopenia; therefore, early nutritional risk screening and prompt intervention are essential [36]. Current guideline panels recommend early oral refeeding as soon as tolerated for mild AP and early enteral nutrition (nasogastric or nasojejunal) for patients who cannot eat or who have predicted severe disease [37]. Early enteral feeding has been associated with fewer infections, a reduced need for surgical interventions, shorter hospital stays, and a lower mortality rate compared to parenteral nutrition [36,38,39]. In older patients, the same preference for enteral over parenteral nutrition applies if the gastrointestinal tract function is undisturbed. However, care must be individualized: baseline nutritional status should be assessed by validated instruments (e.g., using Mini Nutritional Assessment for geriatric patients aged ≥65, and Nutritional Risk Screening 2002 for hospitalized patients) and it is recommended for patients with predicted mild to moderate acute pancreatitis, whereas all patients with predicted severe disease should be assumed to be at nutritional risk; protein requirements should be increased to prevent muscle loss (approximately 1.2–1.5 g/kg/day, if tolerated), and semi-elemental or short-peptide formulas may be considered in patients who develop gastrointestinal intolerance to standard polymeric enteral formulas—manifested by persistent diarrhea, abdominal distension, or clinical signs of malabsorption—as these preparations are more easily digested and absorbed due to their simplified nutrient composition. Additionally, clinicians should monitor for dysphagia and aspiration risk and closely follow tolerance, electrolytes, and organ function [36]. Parenteral nutrition should be considered only when enteral feeding cannot be initiated, is not tolerated or is insufficient to meet the patient’s nutritional needs [16].

### 8.4. ERCP: Indications and Safety

ERCP is indicated in severe AP with cholangitis or persistent biliary obstruction [22]. Numerous studies have demonstrated that ERCP is technically successful and generally safe in elderly populations, including those over 80 or even 90 years of age [40,41]. A recent retrospective study found no significant differences in technical success rates or perioperative complication rates between patients aged ≥90 and those <90 years, with success rates exceeding 96% in both groups [42].

Similarly, another analysis involving patients aged ≥80 reported high success rates with acceptable complication profiles [41]. While older adults experience a lower incidence of post-ERCP pancreatitis compared with younger patients, other complications—such as bleeding and cardiopulmonary events—may occur more frequently [43]. In a comprehensive systematic review and meta-analysis, 69 studies were included. The analysis assessed the incidence of adverse events following ERCP in patients aged ≥65 years. The pooled incidence rates per 1000 ERCPs were: perforation 3.8 (95% CI: 1.8–7.0), pancreatitis 13.1 (95% CI: 11.0–15.5), bleeding 7.7 (95% CI: 5.7–10.1), cholangitis 16.1 (95% CI: 11.7–21.7), cardiopulmonary events 3.7 (95% CI: 1.5–7.6), and mortality 7.1 (95% CI: 5.2–9.4). Compared to younger patients, those aged ≥65 years had a significantly lower risk of post-ERCP pancreatitis (IRR 0.3; 95% CI: 0.3–0.4). However, among older patients, the octogenarians exhibited a 2.4-fold increased risk of mortality (IRR 2.4; 95% CI: 1.3–4.5), while nonagenarians had higher rates of bleeding (IRR 2.4; 95% CI: 1.1–5.2), cardiopulmonary complications (IRR 3.7; 95% CI: 1.0–13.9), and mortality (IRR 3.8; 95% CI: 1.0–14.4). This analysis indicates that while ERCP is generally safe in patients over 65, the patients ≥90 years are at significantly high risk of bleeding, cardiopulmonary complications, and death [43].

### 8.5. Cholecystectomy and Acute Pancreatitis

Cholecystectomy plays crucial role in the prevention of acute biliary pancreatitis (ABP). By removing the gallbladder as the source of gallstones, cholecystectomy reduces biliary obstruction, bile reflux, and pancreatic duct hypertension. Additionally, the procedure mitigates pancreatic autodigestive injury caused by ductal obstruction and attenuates the inflammatory response, which explains the less severe course of AP in patients who have undergone cholecystectomy [44]. At present, surgical removal of the gallbladder due to gallstone cholecystitis represents the most common abdominal procedure among elderly patients [45]. According to the American Gastroenterological Association guidelines, in patients with mild acute gallstone pancreatitis, laparoscopic cholecystectomy is recommended during the same hospitalization, as this significantly reduces the risk of recurrent pancreatitis and related complications. In patients with moderate to severe acute pancreatitis, it is advised to delay cholecystectomy until the resolution of the inflammatory process and clinical stabilization [46]. A total of 30,062 adults with acute biliary pancreatitis (ABP), including 9728 elderly patients (≥70 years), were identified, of whom 13,574 (45%) underwent early cholecystectomy. Among patients younger than 70 years, approximately 49% received early cholecystectomy, compared to 36% of elderly patients. After propensity score matching, younger patients (<70 years) who underwent early cholecystectomy showed significantly lower 90-day mortality (RR 0.16, *p* < 0.001) and 90-day rehospitalization rates (18.5% vs. 34.2%, RR 0.54, *p* < 0.001). Early cholecystectomy was also associated with a reduced risk of acute cholangitis (RR 0.60, *p* < 0.001). In the elderly subgroup, similar benefits were observed, with improved 90-day mortality (1.3% vs. 4.9%, RR 0.26, *p* < 0.001) and lower 90-day rehospitalization rates (29% vs. 39%, RR 0.76, *p* < 0.001). Overall, early cholecystectomy significantly decreases short-term mortality and hospital readmissions in both younger and older patients with acute biliary pancreatitis [47].

## 9. Possible Pathophysiological Mechanisms Underlying Age-Related Differences in Acute Pancreatitis

The differences in clinical course and outcomes of acute pancreatitis (AP) between elderly and younger patients are likely driven by several interrelated pathophysiological mechanisms. With advancing age, the body’s physiological reserve across multiple organ systems diminishes, reducing the capacity to respond to systemic inflammatory stress. This decline in organ resilience makes older individuals more susceptible to shock, multi-organ dysfunction, and higher mortality, particularly in severe cases [8]. Age-related changes in immune function, often described as immunosenescence and inflammaging, further contribute to these vulnerabilities. Older adults exhibit impaired adaptive immunity and dysregulated innate responses, including diminished neutrophil function and altered monocyte activity, resulting in a chronic low-grade inflammatory state. In AP, this immune profile can amplify the initial inflammatory response and limit effective defense against secondary infections, such as those arising from pancreatic necrosis or bacterial translocation [48]. Another important factor is frailty, a multidimensional syndrome characterized by decreased physiological resilience and greater vulnerability to stressors. Patients with frailty exhibit greater susceptibility to severe inflammatory responses and complications, and frailty has been independently associated with higher rates of morbidity and mortality in elderly individuals facing acute illness [49].

## 10. Future Directions

Future research on acute pancreatitis in the elderly could focus on integrating geriatric-specific factors, such as frailty and multimorbidity, into risk stratification. While commonly used scoring systems, including APACHE II, BISAP, and Ranson criteria, incorporate age numerically and clearly reflect the higher risk in older patients, they do not account for these additional geriatric characteristics, which may lead to underestimation or misclassification of disease severity. Optimizing management strategies, particularly fluid resuscitation tailored to elderly patients, as well as clarifying the timing and outcomes of interventional procedures such as ERCP, are further important areas for investigation. Large, prospective, multicenter studies dedicated to elderly populations would provide more robust evidence to guide personalized care and improve clinical outcomes.

## 11. Conclusions

Acute pancreatitis (AP) demonstrates significant age-related differences in epidemiology, etiology, and clinical outcomes. The incidence of AP is considerably higher in older adults compared to younger individuals. While alcohol consumption remains the leading cause in younger patients, biliary etiology—particularly cholelithiasis—is more common in older adults. Furthermore, older individuals tend to experience a more severe disease course, with studies showing that 14.8% of patients aged 65–80 years and 39.6% of those over 80 years develop severe AP, contributing to longer hospital stays, higher rates of complications, and significantly increased mortality (Table 1). In addition to systemic complications, such as organ failure—primarily respiratory and cardiovascular—and infections, elderly patients are also more prone to local complications, including pleural effusions; however, data on age-related differences in other local complications, such as acute peripancreatic fluid collections, pseudocysts, and walled-off necrosis, remain inconsistent. Contributing factors include delayed diagnosis due to atypical clinical presentations and a higher prevalence of comorbidities. Optimal management requires early diagnosis, careful fluid resuscitation with lactated Ringer’s solution under close hemodynamic and renal monitoring. Adequate pain control is also essential. NSAIDs should be selected individually, considering comorbidities and contraindications, and used for the shortest possible duration (up to 10 days) at the lowest effective dose. Early enteral nutrition is preferred over parenteral feeding, and ERCP should be performed when indicated, as it remains safe and effective. Further research is warranted to develop tailored clinical guidelines aimed at improving outcomes in this vulnerable population.

## Figures and Tables

**Table 1:** Summary of studies reporting on the outcomes of acute pancreatitis in patients younger and older than 60 years.

Category	Under 60 Years	60 Years and Older
**Etiology**		
Biliary	19.3–47.6% [6,7]	54.1–89.6% [5,6,7]
Alcohol	10.2–36.2% [5,6]	0–9.5% [5,6]
Hypertriglyceridemia	14.3–39.5% [6,7]	0–5.6% [6,7]
Idiopathic	11.9–37.85% [5,6]	5.3–38.78% [5,6,7]
**Local complications** **(CT/imaging)**		
Acute peripancreatic fluid collection (APFC)	27.2–41.0% [6,7]	34.4–50.9% [6,7]
Acute necrotic collection/Walled-off necrosis (ANC/WON)	1.6–10.0% [6,7]	6.6–36.8% [6,7]
Pancreatic abscess	17.3% (single study; SAP) [13]	13.9% (single study; SAP) [13]
**Multiple organ dysfunction** **syndrome (MODS)**		
MODS incidence	≈0.6% (proxy <65) [6]	≈9.42% (≥80) [6]
**Mortality**		
All-cause mortality (episode/in-hospital or week-5)	Lower: 0.5 [21]; Upper: 7.69 [7]	Lower: 4.59 [5]; Upper: 50.0 [6]
**Length of hospital stay**		
Hospital (days)	N/A	8.70–11.51 [9]

## Data Availability

No new data were created or analyzed in this study.
